# Lignocellulosic Waste Compounds for Pancreatic Lipase Inhibition: Preliminary Extraction by Freon, Obtaining of Proanthocyanidins and Testing on Lipase Activity

**DOI:** 10.3390/metabo13080922

**Published:** 2023-08-07

**Authors:** Anna Andersone, Sarmite Janceva, Liga Lauberte, Jelena Krasilnikova, Natalija Zaharova, Vizma Nikolajeva, Gints Rieksts, Galina Telysheva

**Affiliations:** 1 Latvian State Institute of Wood Chemistry, Dzerbenes 27, LV-1006 Riga, Latvia; natalija.zaharova@gmail.com (N.Z.); telysheva@gmail.com (G.T.); 2 Department of Biochemistry, Riga Stradinš University, Dzirciema 16, LV-1007 Riga, Latvia; liga.lauberte@rsu.lv (L.L.); jelena.krasilnikova@rsu.lv (J.K.); 3 Faculty of Biology, University of Latvia, Jelgavas 1, LV-1004 Riga, Latvia; vizma.nikolajeva@lu.lv; 4 The Institute of Physics, University of Latvia, Miera 32, LV-2169 Salaspils, Latvia; gints.rieksts@inbox.com

**Keywords:** obesity, sea buckthorn, freon R134a, blackcurrant, gooseberries, quince, grapes, polyphenols, proanthocyanidins, lipase inhibitors

## Abstract

The twigs of sea buckthorn, blackcurrant, gooseberries, quince, and grapes were evaluated as a promising source of biologically active compounds—proanthocyanidins (PACs). Sea buckthorn twigs had the highest content of PACs (9.2% on dry biomass). Preliminary pretreatment of biomass with freon R134a did not allow an increase in PACs content in the composition of hydrophilic extract but confirmed the value of freon extract as an antibacterial agent against *P. aeruginosa* and *B. cereus*. The content of PACs was used as an indicator for assessment of the influence of hydrophilic extracts on pancreatic lipase activity. Under normal physiological conditions, in the presence of bile, the extract, which contained 42.4% of PACs was more effective compared to the extract which contained 17.5% of PACs. At all concentrations (0.2–40 mg of sample/g of pancreatic lipase), it inhibited lipase activity by 33%. Purified PACs were the most effective in inhibiting lipase activity (by 36%). However, in pathological physiological conditions (without bile), the opposite effect on lipase activity was observed. Thus, PACs and extracts can be used as inhibitors of pancreatic lipase only under normal physiological conditions.

## 1. Introduction

It was recently proven, based on the example of sea buckthorn, that lignocellulosic biomass of fruit trees/shrubs can be as valuable as fruits in terms of bioactive compounds. Moreover, the content of some of the secondary metabolites is even bigger in their lignocellulosic biomass than in fruits [1]. As all fruit shrubs undergo pruning to a bigger or lesser extent, the volume of waste biomass increases along with increase in berry industrial production volume. Today, this biomass is mostly burned or incorporated into soil directly or through composting. Such a method of biomass application has a small added-value and, additionally, causes greenhouse gas (GHG) emission, since soil microflora induced by such biomass stimulates the production and emission of NO and N_2_O, which have significantly bigger GWP than CO_2_ [2]. Following the authors’ research on sea buckthorn waste biomass, innovative research and comparison of other fruit shrubs’ biomasses is necessary as this opens possibilities for their future applications as a source for separate compounds or extracts complex. Regarding the underutilized lignocellulosic biomass of the range of fruit shrubs cultivated in Europe, berries, which are highly nutritional and reported to have anti-diabetic and weight-reduction properties, were chosen for this study: blackcurrant [3,4], cultivated on a large scale, especially in Poland [5]; gooseberry [6,7], grape [8], and quince [9]. The fruits, pomace, and leaves of the above-mentioned fruit shrubs are used in folk medicine, but only a few publications are available on the phenolic compounds and lipophilic extracts of these types of biomass. Preliminary studies have shown that proanthocyanidins (PACs) are the dominant polyphenols in lignocellulosic biomass [10,11], which are reported to have anti-diabetic, anti-inflammatory, anticancer, antioxidant, cardio-protective, antimicrobial and other health-related properties [11,12]. Previously published data on PACs of the above-mentioned fruit shrub lignocellulosic waste are scarce, so the comparative research of this lignocellulosic biomass was a novelty of this work.

The target PAC properties studied in this research were connected with the anti-diabetic and anti-obesity properties of PACs, since obesity is one of the most influential diseases today. This is mainly caused by excessive consumption of sugar and high fat foods when all consumed energy is not used through physical activity and is stored by the body as fat. According to the World Health Organisation, around 2 billion adults currently are overweight, of whom 650 million are affected by obesity (BMI ≥ 30 kg/m²) [13,14] with side-effects of coronary heart disease, type 2 diabetes, cancers, atherosclerosis, hypertension, hyperlipidemia, dyslipidemia, stroke, and other health problems [15,16,17,18,19,20,21].

A symptomatic solution to prevent obesity could be the action of digestive enzymes [22,23]. Pancreatic lipase is a key enzyme for triglyceride absorption in the small intestine [24]. This enzyme is secreted from the pancreas and hydrolyses triglyceride into glycerol and fatty acids. The suppression of triglyceride absorption by lipase inhibition is one of the additional approaches for preventing obesity [25]. Many people choose medications for effective withdrawal of symptoms and rapid results [26,27]. Currently, in clinical practice, many synthetic drugs are used, characterized by various combinations of components and enzymatic activity. However, the risk of side effects and the cost of these synthetic drugs is high compared to herbal examples [27,28]. Among the commercially available drugs that inhibit the absorption of intestinal fat due to the inhibitory activity of lipase are orlistat (Xenical^®^), lorcaserin (Belviq^®^), phentermine/topiramate (Qsymia^®^), naltrexone/bupropion (Contrave^®^) and liraglutide (Saxenda and Victoza^®^). All these drugs are not safe, as they have such side effects as oily discharge from the rectum, gas, imperative urge to defecate, steatorrhea, increased defecation, and fecal incontinence. In addition, increased blood pressure, high pulse rate, palpitation, dry mouth, headache, insomnia, memory impairment, and paraesthesia have been reported [29].

It was proven that PACs can inhibit digestive enzymes (pancreatic lipase, α-amylase, and trypsin), with different inhibitory potency [27,30]. Since obesity is often accompanied by prolonged infectious deceases and inflammation [31], our hypothesis is that the complex properties of waste-biomass-derived extracts and PACs could be a solution to the complex problems of obesity; therefore, the anti-oxidant and anti-microbial properties of the lipophilic and hydrophilic extracts were also studied. It was shown in our previous research that agro-waste from fruit trees could serve as a raw material for a range of biologically active molecules [12,32].

Sequential extraction allows the obtaining of both non-polar and polar compounds. Lately, extraction by liquified gases, such as supercritical CO_2_, butane, and freons, has started to be developed extensively, mostly for plant material with a high content of lipophilic compounds (orange peels, carrots, sunflower seeds, lavender flowers, etc.) [33,34]. Gas extraction with liquefied hydrocarbons (butane and propane) also reported less decomposition or modification of the compounds [35]. The liquified gases CO_2_ and freon R134a were reported to be efficient for the extraction of cannabis, as the safest and highest-capacity processing methods [36]. It was shown that using a mixture of CO_2_ and R134a solvents can reduce the amount of supercritical fluid necessary [37]. Extraction with R134a does not demand such a high pressure as with supercritical CO_2_ and is generally much cheaper. As an advantage of extraction by fluoro-hydrocarbons, the possibility of changing the yield of extractable compounds by the addition of a co-solvent is also mentioned [38]. Both CO_2_ and freon are approved by the FDA as safe solvents and can replace hexane solvent, which is highly toxic and is banned for use in Europe. Using a closed loop in a freon extractor prevents the R134a global warming effect. The disadvantage of R134a freon extraction is the production stage of the freon from trichloroethylene. Therefore, supercritical CO_2_ extraction seems to be much “greener”. However, CO_2_ extraction demands much more electricity for heating, pressure, cooling, and intensive ventilation to prevent suffocation [39]. CO_2_ capturing increases the fuel needs of an electricity plant by 25–40% [40], and demands amine absorbents [41] which in vivo may form nitrosamines and nitramines, affecting health and the environment; it was found that several of the amines can be highly carcinogenic [41]. Thus, further study would be necessary for the comparison of both extraction methods, which is planned in the future. The innovative freon extraction technology has still undergone very limited research with regard to lignocellulosic biomass, and in this study this was evaluated. The influence of freon extraction conditions on further extraction of hydrophilic compounds by water and ethanol/water solutions was studied.

Based on the analysis performed, the present study aimed to obtain the lipophilic and hydrophilic extracts and PACs from the twigs of five types of fruit trees/shrubs (sea buckthorn, blackcurrant, gooseberries, quince, and grapes), widespread in the Baltic States and Europe, the chemical characterization of the obtained extracts, and the study of the effect of extracts and isolated PACs on the activity of the pancreatic lipase in normal and pathological patient conditions. The anti-microbial properties of lipophilic compounds obtained by hexane and freon were tested for preliminary validation of the concept of natural materials-based complex solutions for obesity patients. To the best of the authors’ knowledge, this is the first comparative study of the above-mentioned lignocellulosic biomass. Thus, the novelties of the present study are the usage of the above-mentioned lignocellulosic agro-waste as a source of extracts and PACs; evaluation of freon extraction as a method to obtain polar and semi-polar compounds from lignocellulosic biomass; study of the effect of co-solvent; anti-microbial properties of lipophilic extracts; and analysis of the influence of hydrophilic extracts and PACs on lipase activity.

## 2. Materials and Methods

### 2.1. Collection of Plant Material

The twigs of sea buckthorn (*Hippopae rhamnoides* L.), cultivar ‘Maria Bruvele’, Institute of Wood Chemistry (IWC) sample storage No. 91/03-20/146-22, were collected from the sea buckthorn plantation area in Seme parish, Tukums county of Latvia (DD: 57.1444093, 23.108156); blackcurrant (*Ribes nigrum* L.), cultivar ‘Selechenskaja’, IWC No. 97/03-20/146-22; gooseberries (*Grossulariaceae* family), cultivar ‘Kuršu Dzintars’, IWC No. 98/03-20/146-22; quince (*Chaenomeles japonica*), cultivar ‘Rasa’, IWC No. 99/03-20/146-22; and grapes (*Vitis vinifera*), cultivar ‘Zilga’, IWC No. 100/03-20/146-22, were collected from the fruit-tree/shrub plantation area of Baldone parish, Kekava county of Latvia (Decimal degrees (DD): 56.82065, 24.27653). Twigs were cut in September 2022. All biomass samples are deposited in the Institute of Wood Chemistry (IWC), room 239, at the freezer, and available for analyses. The twigs were dried at 20–25 °C temperature, leaves were removed, and the twigs were grounded with a mill (Retsch SM100, RETSCH, Haan, Germany). The grounded twigs biomass (further in the text—biomass) was stored at −8 °C.

### 2.2. Reagents

The solvents (high purity), DPPH^•^ (2,2-diphenyl-1-picrylhydrazyl), ABTS^+•^ (2,2′-azinobis(3-ethylbenzothiazoline-6-sulfonic acid), the reference antioxidants Trolox (6-hydroxy-2,5,7,8-tetramethylchroman-2-carboxylic acid), as well as analytical standards gallic acid (purity ≥ 97.5%) and procyanidin B2 (purity ≥ 90%) were purchased from Sigma-Aldrich (St. Louis, MO, USA). Reference microbial strains: *S. aureus* MSCL 3340, *P. aeruginosa* MSCL 3314, *E. coli* MSCL 332, *B. cereus* MSCL 330 were received from the Microbial Strain Collection of Latvia (MSCL, Riga, Latvia).

### 2.3. Isolation of the Extracts from Biomass

**Figure 1:**
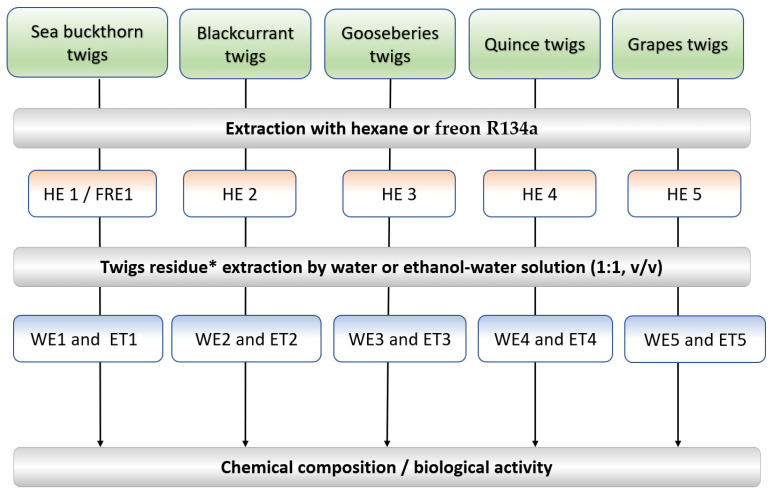
Principal schema of extracts isolation from biomass: * biomass residue after extraction with hexane; H-hexane extract; WE-water extract; ET-50% EtOH extract.

The scheme of extracts’ isolation from dry biomass (DB) is shown in Figure 1.

#### 2.3.1. Lipophilic Extract

Lipophilic extracts (HE 1-HE 5, Figure 1) from biomass were isolated by extraction at 50–60 °C for 40 min using n-hexane as a solvent. The extracts after hexane evaporation were dried at 40 °C to yield a dry extract (DE). The yield of the extracts is presented as a percentage based on DB. The confidence interval (CI) for the results did not exceed 3% at α = 0.05).

#### 2.3.2. Semipolar Extracts

Semipolar extract (FRE 1, Figure 1) from DB of sea buckthorn (with/without co-solvent (ethanol) was isolated by ozone-friendly 1,1,1,2-tetrafluoroethane (freon R134a) in a closed system under a pressure of 4.0–4.3 Bar and a temperature of 17–19 °C. The yield of the extracts is presented as a percentage based on DB. The CI for the results did not exceed 3% at α = 0.05).

#### 2.3.3. Hydrophilic Extracts

Hydrophilic extracts (WE 1-WE 5 and ET 1-ET 5, Figure 1) from the DB were isolated by extraction at 60 °C for 40 min (20 min × 2 times) using distilled water and 50% EtOH solution (ethanol: distilled water, 1:1, *v*/*v*). The extracts, after ethanol evaporation, were freeze-dried to yield a DE. The yield of the DE is presented as a percentage based on ODB. The CI for the results did not exceed 3% at α = 0.05.

### 2.4. Determination of Total Phenol Content in the Extracts

The total polyphenol (TP) content in the extracts (WE 1-WE 5 and ET 1-ET 5, Figure 1) was quantified by the Folin–Ciocalteu method using gallic acid (GA) as a reference compound. An aliquot (1 mL) of the extract was transferred into the test tube, 5 mL of Folin–Ciocalteu reagent and 4 mL 7% aqueous sodium carbonate solution were added; the tube was vortexed and placed into a water bath at 30 °C for 1 h. The absorbance of the mixture was then recorded at 760 nm with a UV/VIS spectrometer Lambda 650 (Perkin Elmer, Inc., Waltham, MA, USA) against a blank containing 1 mL of extraction solvent. The content of TP was calculated as a GA equivalent (GAE) from the standard curve and expressed as g GAE·100 g^−1^ DE. All measurements were carried out in triplicate. The CI for the results did not exceed 3% at α = 0.05.

### 2.5. Determination of PACs Content in the Extracts

The content of PACs in extracts (WE 1-WE 5 and ET 1-ET 5) and purified PACs fraction were measured by the butanol-HCl method using procyanidin dimer B2 as a reference compound. An aliquot (1 mL) of the extract was transferred into the test tube, 6 mL of acid butanol (5% (*v*/*v*) concentrated HCl in n-butanol) and 0.2 mL of iron reagent (*w*/*v*) (FeNH_4_(SO_4_)_2_∙12 H_2_O in 2 N HCl) were added; the tube was vortexed and placed into water bath at 80 °C for 50 min. The absorbance of the mixture was then recorded at 550 nm with a UV/VIS spectrometer Lambda 650 (Perkin Elmer, Inc., Waltham, MA, USA) against a blank containing 1 mL of extraction solvent. Each sample was analyzed in triplicate, and assay results were expressed as a percentage per DE. The CI for the results did not exceed 3% at α = 0.05).

### 2.6. Determination of Total Flavonoid Content in Extracts

Next, 5–10 mg of the DE was dissolved in 25 mL of 96% ethanol. Then 0.4 mL of the extract solutions were added to a 10 mL test tube containing water (2 mL). After that, 5% sodium nitrite (NaNO_2_) solution (0.12 mL) was added and incubated for 5 min at room temperature, after which 10% aluminium nitrate solution (0.24 mL) was added to the mixture. After 6 min, 1 mol·L^−1^ sodium hydroxide (0.8 mL) was added. The absorbance was measured at 420 nm, and the result was expressed as mg of quercetin equivalents per g of dry weight (DW).

### 2.7. Determination of Total Tannin Content in Extract

All extract aliquots were divided into two parts. To one part, phosphomolybdotungstic reagent was added. After pH adjustment with sodium carbonate solution and 30 min reaction time, absorbance was measured at 760 nm using a UV/VIS spectrometer Lambda 650 (Perkin Elmer, Inc., Waltham, MA, USA). The second part of the extract was shaken with hide powder CRS and filtered. The phosphomolybdotungstic reagent was added to the filtrate, and after pH adjustment and 30 min of reaction time, absorbance was measured at 760 nm. As a standard substance, pyrogallol was used after a reaction with a phosphomolybdotungstic reagent. Polyphenols adsorbed by hide powder (=tannin content) were calculated by subtracting the unabsorbed polyphenols from the total polyphenols, each referring to the absorption of the standard substance. The tannin content was expressed as a percentage per DE. The CI for the results did not exceed 3% at α = 0.05).

### 2.8. Determination of PAC Composition by LC-UV-ESI-QTOF MS Analysis

Dry purified PAC fraction was dissolved in aqueous methanol (*v*/*v*; 2:8) with an approximate concentration of 0.1 mg·mL^−1^ after which it was filtered and then used for TOF/MS experiments. The TOF-MS spectra of PACs were recorded with Waters Acquity UPLC HClass with a PDA detector and Micromass QuattroMicro Mass spectrometer (Waters Corp., Milford, MA, USA) using the Acquity UPLC BEH Amide column (1.7 µm, 3.0 × 100 mm).

### 2.9. Determination of Lipophilic Extract Composition by GC-MS Analysis

Lipophilic extracts (HE 1 and FRE 1) were analyzed by GC-MS chromatography using Shimadzu GC/MS/FID-QP ULTRA 2010 apparatus (Shimadzu, Kyoto, Japan), a capillary column RTX-1701 (Restec, Metairie, LA, USA) and a 60 m × 0.25 mm × 0.25 mm film (an injector temperature of 250 °C, an ion source with EI of 70 eV, carrier gas helium at the flow rate of 1 mL min^−1^ and a split ratio of 1:30). Dry extracts (residual moisture content <1%) were dissolved in hexane (*w*/*w* 1:10) with an approximate concentration of 0.1 g/g _hexane_ and filtered (Nylon filter, 0.45 μm pore size), after which they were used for GC-MS experiments. The oven program: 1 min isothermal at 60 °C, followed by 6 °C min^−1^ to 270 °C, and the final hold at 270 °C for 10 min. The mass spectrometer was operated in electron impact mode using 70 eV electron energy. The identification of the individual compounds was performed based on GC/MS using Library MS NIST 11 and NIST 11s, whereas the relative area of the peak of individual compounds was calculated using Shimadzu software based on GC/FID data. The summed molar areas of the relevant peaks were normalized to 100%, and the data for four repetitive pyrolysis experiments was averaged. The variation coefficient of measurement was ≤5%.

### 2.10. Determination of Radical Scavenging Activity

#### 2.10.1. DPPH^•^ (2,2-Diphenyl-1-picrylhydrazyl Radical) Assay

The dry extracts (HE 1L, FRE1, WE 1-WE 5 and ET 1-ET 5) and purified PAC fraction were tested for their radical scavenging activity against the 2,2-diphenyl-1-picrylhydrazyl (DPPH^•^) using a UV/VIS spectrometer Lambda 650 (Perkin Elmer, Shelton, CT, USA). The DPPH^•^ assay was measured according to the procedures described by Dizhbite et al. [42]. A range of different concentrations of the obtained DE in DMSO was prepared. The absorbance at 515 nm was measured 15 min after the mixing of 30 µL of extract (or antioxidant standard) with 3.0 mL DPPH^•^ (1·10–4 mol∙L^−1^) solution. DMSO was used as a control and Trolox as a reference antioxidant standard. CI ≤ 0.3 mg·L^−1^.

#### 2.10.2. ABTS^+•^ (2,2′-Azinobis (3-Ethylbenzothiazoline-6-sulfonic Acid) Assay

The dry extracts (WE 1-WE 5 and ET 1-ET 5) and purified PAC fraction were tested for their radical scavenging activity against the 2,2′-azinobis (3-ethylbenzothiazoline-6-sulfonic acid (ABTS^+•^) using UV/VIS spectrometer Lambda 650 (Perkin Elmer, Shelton, CT, USA). ABTS^+•^ was produced by the reaction of 2 mmol·L^−1^ ABTS stock solution with 70 mmol·L^−1^ potassium persulfate (K_2_S_2_O_8_), allowing the mixture to stand in the dark at room temperature for 12–16 h before use. The ABTS^+^ solution (stable for 2 days) was diluted with phosphate-buffered saline (pH 7.4) to an absorbance of 0.80 ± 0.02 at 734 nm. The absorbance at 734 nm was investigated 10 min after the mixing of 30 µL of extract (or purified PACs fraction and antioxidant standard—Trolox), diluted in DMSO of five different concentrations with 3.0 mL ABTS^+•^ solution. DMSO was used as a control and Trolox as the antioxidant standard. CI ≤ 0.3 mg·L^−1^.

### 2.11. Antimicrobial Activity of Lipophilic Extracts

Minimum inhibitory concentration (MIC) determination by microtiter broth dilution method was used. Stock solutions of the respective plant extracts were prepared in 10 mL microcentrifuge tubes by dissolving dry plant extract in dimethyl-sulfoxide (DMSO) to a final concentration of 100 mg·mL^−1^. The serial dilutions from the stock solution were made, ranging from 50 mg·mL^−1^ to 0.01 mg·mL^−1^, using Mueller–Hinton broth (Becton Dickinson, Sparks, MD, USA) in 96-well microplates. The microbial suspension containing approximately 5 × 10^5^ colony-forming units (CFU)/mL was prepared from a 24 h culture plate. From this suspension, 100 μL was inoculated into each well. The microtiter plates were incubated at 37 °C, for 24 h. After incubation, 40 μL of a 0.4 mg·mL^−1^ solution was added to each well as an indicator of microbial growth. The plates were incubated for 30 min and the MIC values were determined.

Minimum bactericidal concentration (MBC) was recorded as the lowest concentration of an antimicrobial substance that reduces the viability of the initial microorganism inoculation by ≥99.9% after 24 h incubation at 37 °C. MBC was evaluated for extracts and purified PACs fraction. 10 μL were taken from the well obtained from the MIC experiment (MIC value), as well as two wells above the MIC value well, and spread on Mueller–Hinton agar plates. The number of colonies was counted after 18–24 h of incubation at 37 °C. The concentration of sample that produces <10 colonies was considered as MBC value. CI ≤ 0.3 mg·L^−1^.

### 2.12. Determination of the Influence of Extracts and Purified PACs on Lipase Activity

The test tubes were prepared composed of 4.0 mL milk (3.8% fat), 1 mL pancreatic solution (pancreatin from porcine pancreas, Sigma-Aldrich), 1 mL bile (bile extract porcine, Sigma-Aldrich), and 1 mL phenolphthalein. Orlistat (purity ≥98%, Sigma-Aldrich), a known inhibitor of pancreatic lipase, was used as a reference compound. The first test tube was used as a control tube without the investigated components (PACs, extracts, and reference). To the following tubes, 100–2000 μL of the analyzed sample (PACs, extract or Orlistat) at the concentrations of 2, 20, and 200 mg·L^−1^ (0.2–400 mg of sample/g of pancreatic lipase—further in the text—mg·g^−1^ PL) were added. All tubes were put in the incubator for 40 min at 38 °C. Afterwards, each solution from the tubes was titrated with 0.1 N NaOH solution until the colour changed into yellowish-brown, and the needed amount of NaOH was determined. CI ≤ 0.3 mg· g^−1^ PL.

### 2.13. Statistical Analysis

All measurements were conducted in triplicate and the results were presented as the mean value. Statistical analyses were performed using Microsoft Excel 2016, version No. 16. CI for a mean using Student’s *T* distribution was calculated at a significance level of 5% (α = 0.05).

## 3. Results and Discussion

### 3.1. Lipophilic and Hydrophilic Extracts from Fruit-Tree Biomass

The yields of lipophilic extracts obtained with hexane from the entire studied biomass were quite close, but statistically different, and varied from 0.92 to 1.44% per DB. The yields of hydrophilic extracts from the same biomass, obtained using distilled water as the most environmentally friendly solvent, differed statistically significantly and varied from 7.1 to 15.7% per DB. With 50% EtOH, the extracts yield increased significantly, suggesting that the extractives are more soluble in the ethanol–water suspension. The yields of hydrophilic extracts obtained with 50% EtOH from the entire biomass under study were: sea buckthorn 21.7% > quince 14.3% > grapes 13.9% > black currant 13.5% > gooseberry 7.5% (Table 1). The biomass of sea buckthorn differs from the other studied tree species not only by the highest total yield of hydrophilic extracts (21.7% per DB), but also by the high content of polyphenols (48.6 g GAE·100 g^−1^ DE).

Gooseberry biomass had the lowest yield of hydrophilic extracts (7.1% per DB by distilled water and 7.5% per DB by 50% EtOH). Polyphenols played the main role in the biological activity of all hydrophilic extracts. The amount of them in the 50% EtOH hydrophilic extracts was higher and varied from 30.3 to 48.6 g GAE·100 g^−1^ DE. A comparative analysis of the solvent dependence on the yield of polyphenols and between biomasses is shown in Table 2.

The content of TP of sea buckthorn was very similar in years 2020 and 2022, considering the CI (e.g., for 50% EtOH extract: 48.1 ± 0.2 g GAE·100 g^−1^ extract in 2020 [12] against 48.6 ± 0.2 g GAE·100 g^−1^ DE in 2022). The literature data on TP of twigs of the other biomasses under study is very limited. Gooseberry berries were reported to contain TP in the amount of around 3 g GAE·100 g^−1^ biomass [43], which correlates well with our data calculated on biomass (around 2.9 g GAE·100 g^−1^ biomass for 50% EtOH extract). Quince berries were reported to be rich in PACs [44]. But information on lignocellulosic biomass is absent.

Tannins are the dominant polyphenols in all hydrophilic extract compositions. The number of PACs, also known as condensed tannins, from the total amount of tannins ranged from 54 to 83% per DE. The highest content of PACs was observed for sea buckthorn 50% EtOH extracts (42.4% per DE). However, 50% EtOH extract from quince biomass was richer than sea buckthorn (4.9 g RU·100 g^−1^ per DE) in total flavonoid content (12.6 g RU·100 g^−1^ DW). All these polyphenols are biologically active natural antioxidants and antimicrobial agents, which can be used as ingredients in the formulation of different medications. The synergistic effect of the polyphenolic mixtures additionally leads to the simultaneous action on various disease pathways, which consequently contributes to a faster and more effective treatment outcome.

It is known that polyphenols are predominantly present in glycosylated forms. This is the main reason for their low absorption in the stomach, since only aglycones and some glucosides can be absorbed in the small intestine, and the rest are absorbed in the large intestine. The effectiveness of polyphenols absorbed in the colon reaches only 15–20% of the total content of polyphenols absorbed in the intestine. Thus, glucosides in dietary sources of polyphenols provide faster and more efficient absorption of polyphenols [45].

**Figure 2:**
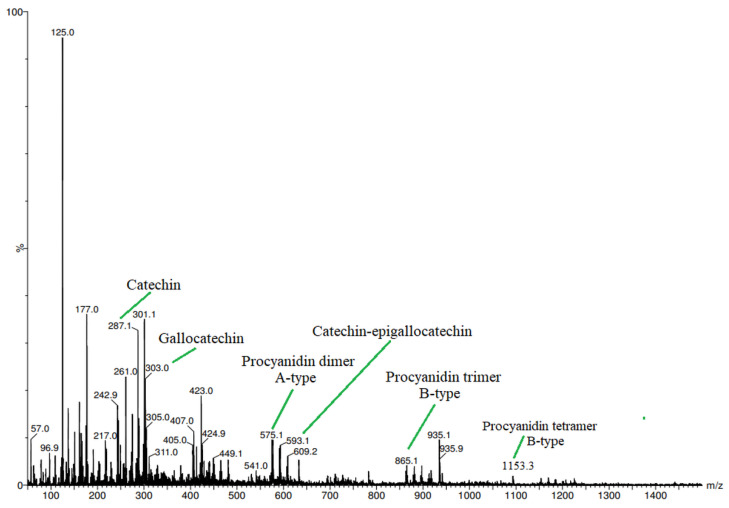
LC-UV-ESI-QTOF-MS analysis of PACs from sea buckthorn biomass.

In order to reduce the number of polyphenolic glycosides and free sugars, fractionation of the extract was carried out using Sephadex® LH-20 (Cytiva, Uppsala, Sweden). The results were obtained in two fractions: a fraction consisting of low molecular polyphenols and their glycosides and free sugar, and a fraction consisting of PACs. The composition of PACs (Figure 2) was determined by LC-UV-ESI-QTOF MS analysis.

### 3.2. Sea Buckthorn Biomass Extraction by Freon R134a

Evaluation of pre-purification of biomass from impurities was carried out using 1,1,1,2-tetrafluoroethane (Freon R134a). Based on the highest content of PACs in the extract (Table 2), the sea buckthorn was used for analysis. To compare with the extraction by hexane, the extraction with freon was carried out in a closed system at 17–19 °C from 8 to 24 h. The yield of freon extract (1.6% per DB) was insignificantly higher, but the composition of extracts obtained differed. When adding a co-solvent (ethanol) from 5 to 10% on dry biomass and carrying out the extraction without freon circulation, but by maceration for 36 h at 17–19 °C, two extracts were separately obtained with a yield of 1.8 and 2.4% per DB. Based on the GC analysis data, the freon extract was richer in total aliphatic and cyclic monomers. The hexane extract was richer in total acid/ester content. The changes in chemical composition between hexane extract and freon extract of sea buckthorn is shown in Table 3.

Since this is the first research on freon extraction from twigs, there were study limitations consisting of seasonal and geographical changes in lipophilic compound content, which have to be further studied. The other types of freon (e.g., R1234yf or R1234ze) also have to be evaluated for the extraction. Although freon R134a is used in a close loop and has 0 ozone depletion, it has a high GHP (1300 vs. 7 vs. of the R1234ze) and its production and application could be banned after a while.

Sea buckthorn biomass residue, after freon extraction with/without co-solvent, was extracted by 50% EtOH. The yield of hydrophilic extracts from all biomass residues slightly decreased. Small changes were seen only in the reduced content of polyphenols in 50% EtOH extract (Table 4).

### 3.3. LC-UV-ESI-QTOF-MS Analysis of Proanthocyanidins Fraction from Sea Buckthorn Hydrophilic Extract

**Figure 3:**
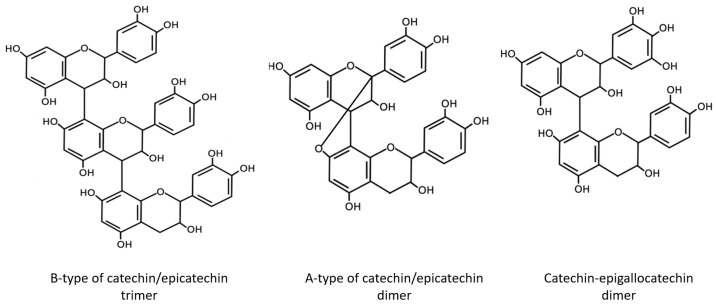
Chemical structures of identified compounds of PACs composition.

According to *LC-UV-ESI-QTOF-MS* analysis (Figure 2), the purified PAC fraction consists of A-type of catechin/epicatechin dimer (*m*/*z* 575), B-type of catechin/epicatechin trimer (*m*/*z* 865), gallo-catechin units (*m*/*z* 303), and catechin-epigallocatechin dimer (*m*/*z* 593) (Figure 3).

### 3.4. Radical Scavenging Activity of Extracts and Purified PACs

The radical scavenging activity of all biomass extracts was evaluated by ABTS^+^ and DPPH^•^ assays. In comparison to Trolox as a reference, which is a water-soluble derivative of vitamin E (IC_50_ = 4.0 mg L^−1^ in ABTS^+•^ test and IC_50_ = 4.7 mg L^−1^ in DPPH^•^ test), the 50% EtOH extracts from sea buckthorn and quince biomass showed the highest radical scavenging activity (IC_50_ = 3.2 and 3.5 mg L^−1^ in ABTS^+•^ test and IC_50_ = 6.1 and 6.6 mg L^−1^ in DPPH^•^ test, respectively) (Table 5).

Due to the low content of polyphenols in blackcurrant, gooseberries, and grape extracts, the radical-scavenging activity was also low. Previous research has shown that purified PACs are powerful antioxidants. The radical scavenging activity of PAC fraction with PACs content 92.1% on dry weight (by Buthanol-HCl assay), was 2.3–2.7 times higher (IC_50_ = 1.4 mg·L^−1^ in ABTS^+•^ test and IC_50_ = 2.3 mg·L^−1^ in DPPH^•^ test) than 50% EtOH extract from sea buckthorn (IC_50_ = 3.2 mg·L^−1^ in ABTS^+•^ test and IC_50_ = 6.1 mg·L^−1^ in DPPH^•^ test). The results for sea buckthorn twigs-derived PACs correlated well with the antioxidant activity of sea buckthorn twigs-derived PACs in year 2020: IC_50_ = 2.6 mg·L^−1^ in DPPH^•^ test, with CI = 0.1 at α = 0.05 [23]. Biomass of the other plant origin has not been previously studied. Freon and hexane extract at a concentration of 30 mg·L^−1^ did not show the ability to inhibit DPPH and ABTS radicals.

### 3.5. Antimicrobial Activity of Lipophilic Extracts from Sea Buckthorn Biomass

Since obesity has a risk of recurrent soft-tissue infections or prolonged hospitalization [46], the antimicrobial activity of lipophilic extracts was tested with Gram-negative and Gram-positive bacteria (Table 6). The results showed that the antimicrobial activity of both extracts was different; for example, according to the MBC value, which showed the complete inhibition of bacteria, the freon extract is more effective against *P. aeruginosa* and *B. cereus.* The hexane extract was more effective against *E. coli* and *S. aureus* bacteria.

### 3.6. Inhibitory Effects of PACs and Other Polyphenol-Containing Samples on Pancreatic Lipase Activity In Vitro

Our previous studies have shown that the bioactivity of PACs was strongly influenced by the degree of purity of the isolated PACs. It was also pointed out that the biological activity of PACs is higher than that of monomeric flavan-3-ols, because as the number of monomeric units bound in oligomeric or polymeric PACs increases, the number of hydroxyl groups also increases, which can donate electrons to neutralize free radicals [47]. The degree of polymerization (DP) appears to affect the ability of PACs to inhibit enzymes in the same way. The relationship between PACs’ structure and function still remains a controversial issue; although there is evidence to support that DP must be high to increase their biological activity, other authors suggest that PACs must have a low DP range in which their biological activity is highest. For example, oligomeric PACs from apples with DP = 5 were good inhibitors of pancreatic lipase activity but their inhibitory activity decreased as their DP increased to decamers [48]. Oligomeric PACs with DP = 2–10 isolated from cocoa beans (Theobroma cacao) also had the highest inhibitory activity (compared to polymeric fractions) over pancreatic amylase, lipase, and phospholipase A2 [49]. Similar effects have been found for PACs from rowan (Sorbus aucuparia): it was found that PAC fraction (containing oligomeric and polymeric PACs) was a better inhibitor of pancreatic α-amylase than polymeric PAC-enriched fractions, although both were effective as enzyme inhibitors [47]. This confimed our previous investigation of PACs from deciduous trees’ biomass as inhibitors of α-amylase [10].

**Figure 4:**
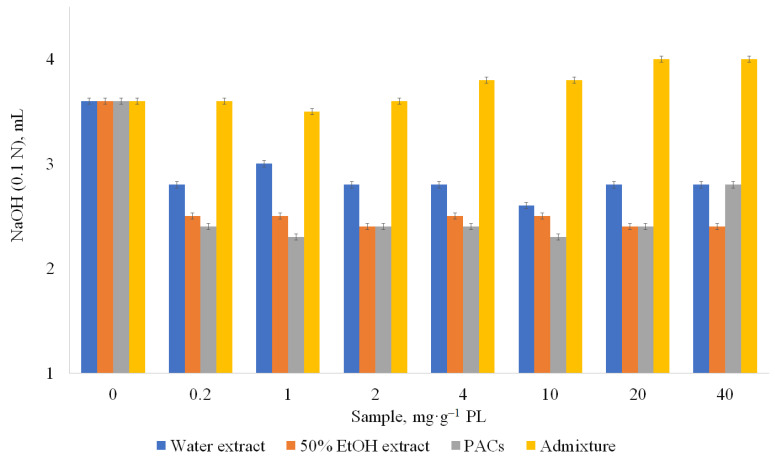
Influence of samples on pancreatic lipase (PL) activity in the presence of bile (normal physiological conditions). CI ≤ 0.3 mg·g^−1^ PL.

Under normal physiological conditions (in the presence of bile), all hydrophilic extracts of sea buckthorn at all concentrations (0.2–40 mg·g^−1^ PL) showed significant inhibition of pancreatic lipase activity. Already at the amount of 0.2 mg of water extract containing 43.4 ± 0.4 g GAE·100 g^−1^ of polyphenols and 17.5 ± 0.1% of PACs, the lipase activity decreased by 22%. Further gradual increase of the amount of the water extract from 0.2 mg·g^−1^ PL to 40 mg·g^−1^ PL gave approximately the same lipase inhibition, within the confidence interval. The dependence of lipase activity inhibition on the content of PACs in the extract has also been established: the higher the content of PACs in the extract, the higher the percentage of inhibition. An extract containing 48.6 ± 0.2 g GAE·100 g^−1^ and 42.4 ± 0.3% PACs at the amount of 0.2 mg·g^−1^ PL to 40 mg·g^−1^ PL inhibited lipase activity by 33%. A PACs fraction (PACs content in extract 92.1% on dry weight) was the best pancreatic lipase inhibitor; at a concentration 1 mg·g^−1^ PL, pancreatic lipase activity decreased by 36%. At the same concentration, Orlistat was 1.4 times more effective (inhibited lipase activity by 52%). However, it has to be considered that Orlistat has side effects, described above in the Introduction. At the concentrations of PACs until 20 mg·g^−1^ PL, lipase inhibition was similar in inhibited value taking into account confidence interval. Therefore, in practical applications, the concentration 0.2–1 mg·g^−1^ PL is sufficient for lipase inhibition and there is no need to increase the concentration. When comparing all tested samples, the fraction of the extract after PAC separation (admixture) was not effective as a pancreatic lipase inhibitor, and at a concentration of 20 mg·g^−1^ PL contributed to the increase of lipase activity (by 6–11%) (Figure 4). This confirms that PACs are the main active compounds inhibiting lipase activity in studied concentrations.

**Figure 5:**
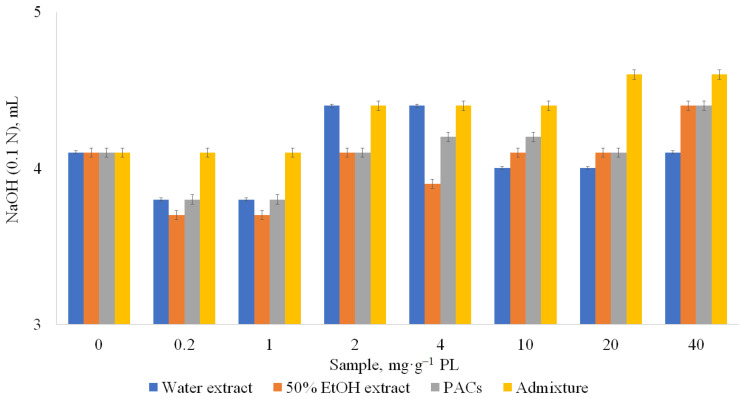
Influence of samples on pancreatic lipase (PL) activity without bile (pathological conditions). CI ≤ 0.3 mg·g^−1^ PL.

Under pathological conditions (without a bile), a similar tendency was observed for lipase inhibition in the 50% EtOH extracts and purified PACs fraction at 0.2 and 1 mg·g^−1^ PL; however, already at 2 mg·g^−1^ PL, pancreatic lipase activity activation was observed (Figure 5).

In the PAC-free extract (admixture), the lipase activity under pathological conditions was higher (10–15%) compared to the results under normal conditions (6–11%). At the amount of samples’ of 0.2 and 1 mg·g^−1^ PL, PACs and extracts showed slight lipase inhibition activity. However, at 2 mg·g^−1^ PL almost all samples showed the neutral or lipase activation effects.

**Figure 6:**
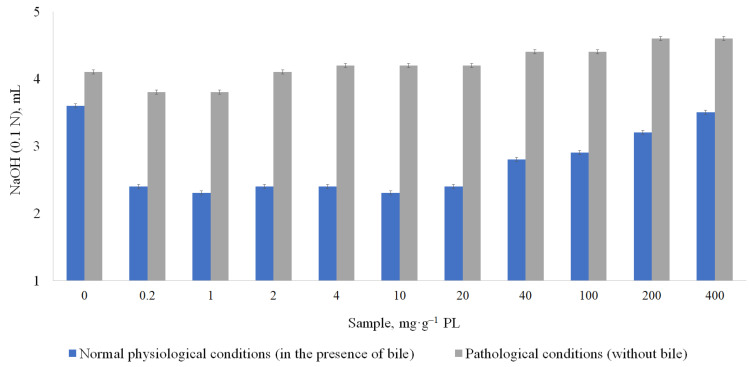
Influence of purified PACs on pancreatic lipase activity. CI ≤ 0.3 mg·g^−1^ PL.

The purified PACs were further tested at the amount of 0.2–400 mg·g^−1^ PL. The purified PAC sample at the amount of 2–400 mg·g^−1^ PL had the activation tendency of lipase activity in pathological conditions (Figure 6).

Authors’ earlier studies showed that PACs are the main component in inhibiting the activity of digestive enzymes in certain concentrations. Under normal conditions, PACs were the most effective at the amount 0.2–20 mg·g^−1^ PL, inhibiting lipase activity by 33–36%. Under pathological conditions, PACs in all concentrations from 2 to 400 mg·g^−1^ PL were lipase action activators.

Future perspectives are the evaluation of the influence of the PACs’ composition on the lipase activity (structure-activity relationship) and clinical studies, which are missing for almost all discovered lipase inhibitors.

## 4. Conclusions

Several fruit shrubs pruning plant material was evaluated and compared for the first time. It was shown that the lignocellulosic biomass originating from agro-waste of fruit shrubs is a source of valuable metabolites. Innovative freon extraction of non-polar and semi-polar compounds, as well as the influence of EtOH as co-solvent on further extraction of hydrophilic compounds, were tested. Lipophilic extracts obtained by freon had a different composition than those obtained by hexane and had a low antioxidant activity (IC_50_ > 30 mg·L^−1^), but showed anti-microbial activity. The freon extract was more effective against *P. aeruginosa* and *B. cereus*, but the hexane extract was more effective against *E. coli* and *S. aureus* bacteria.

The 50% EtOH extracts from sea buckthorn and quince biomass showed the highest radical scavenging capability among all the extracts, but it was still lower than for purified PACs. The sea buckthorn PACs and 50% EtOH extracts had the strongest inhibiting effect on pancreatic lipase (33–36%) in normal physiological conditions. In pathological conditions, both extracts, as well as PACs, tended to slightly increase lipase activity.

Lignocellulosic biomass-derived PACs’ influence on pancreatic lipase proved in the study, along with PACs’ and the lipophilic extracts’ anti-microbial activity, can be used in complex treatments for obesity and its accompanying microbial infections. It has to be pointed out that due to inhibition of lipase, absorption of fat-soluble vitamins and nutrients is inhibited as well. Thus, lipase inhibition could be a temporary solution in fighting obesity, when patient’s pathological molecular mechanisms already developed and it’s not easy to change them just by transition to healthier lifestyle.

## Figures and Tables

**Table 1:** The yield of lipophilic and hydrophilic extracts from fruit trees biomass, %/DB.

Solvents	Sea Buckthorn	Blackcurrant	Gooseberries	Quince	Grapes
Hexane	1.44 ± 0.03	0.92 ± 0.01	1.18 ± 0.01	1.02 ± 0.03	1.16 ± 0.02
50% EtOH (*v*/*v*)	21.71 ± 0.03	13.52 ± 0.03	7.50 ± 0.03	14.29 ± 0.03	13.88 ± 0.02
Water	15.73 ± 0.05	10.77 ± 0.04	7.08 ± 0.05	13.79 ± 0.03	11.71 ± 0.03

**Table 2:** The composition of hydrophilic extracts obtained from fruit tree biomass.

Biomass	TT Content in Extract, % per DE	TP Content in Extract, g GAE·100 g^−1^ DE	PACs Content in Extract, % per DE	TF Content in Extract, g RU·100 g^−1^ DE
	Water extracts			
Sea buckthorn	32.2 ± 0.3	38.4 ± 0.4	17.5 ± 0.1	2.7 ± 0.1
Blackcurrant	14.8 ± 0.2	32.1 ± 0.3	10.6 ± 0.3	4.6 ± 0.2
Gooseberries	10.7 ± 0.5	22.7 ± 0.3	9.0 ± 0.2	2.2 ± 0.3
Quince	18.3 ± 0.3	31.2 ± 0.1	14.0 ± 0.4	9.9 ± 0.1
Grapes	19.2 ± 0.2	18.6 ± 0.5	10.6 ± 0.5	2.5 ± 0.3
	50% EtOH extracts			
Sea buckthorn	44.1 ± 0.5	48.6 ± 0.2	42.4 ± 0.3	4.9 ± 0.1
Blackcurrant	38.2 ± 0.2	33.6 ± 0.1	14.2 ± 0.2	5.1 ± 0.1
Gooseberries	12.6 ± 0.4	38.9 ± 0.2	10.2 ± 0.3	4.2 ± 0.1
Quince	22.5 ± 0.3	42.2 ± 0.2	18.7 ± 0.2	12.6 ± 0.2
Grapes	16.9 ± 0.4	30.3 ± 0.1	11.4 ± 0.2	3.1 ± 0.1

**Table 3:** Comparison between extracts obtained by hexane and freon.

Identified Compounds Group	Freon Extract, % rel	Hexane Extract, % rel
Total acid/ester	30.3	55.7
Total aliphatic and cyclic monomers	43.1	29.9

**Table 4:** Influence of freon on hydrophilic extracts yield and composition.

Sea Buckthorn Biomass	Yield of 50% EtOH Extract, % per DB	TP Content in Extract, g GAE·100 g^−1^ DE	PACs Content in Extract, % per DE
Residue after hexane extraction	21.71 ± 0.03	48.6 ± 0.2	42.4 ± 0.3
Residue 1 (without co-solvent)	20.36 ± 0.02	47.2 ± 0.3	42.2 ± 0.3
Residue 2 (5% of co-solvent)	20.44 ± 0.02	46.8 ± 0.2	41.9 ± 0.3
Residue 3 (10% of co-solvent)	19.98 ± 0.03	46.2 ±0.3	42.5 ± 0.3

**Table 5:** Radical scavenging activity of lipophilic and hydrophilic extracts.

Solvent	Biomass	TP Content in Extract, g GAE·100 g^−1^ DE	IC_50_, mg·L^−1^ by DPPH Test, CI ≤ 0.3 mg·L^−1^	IC_50_, mg·L^−1^ by ABTS Test, CI ≤ 0.3 mg·L^−1^
Hexane	Sea buckthorn	n.d.	>30	-
Freon	Sea buckthorn	n.d.	>30	-
Water	Sea buckthorn	38.4 ± 0.4	9.8	5.6
	Blackcurrant	32.1 ± 0.3	14.1	7.5
	Gooseberries	22.7 ± 0.3	16.6	8.2
	Quince	31.2 ± 0.1	14.4	7.9
	Grapes	18.6 ± 0.5	22.1	10.6
50% EtOH	Sea buckthorn	48.6 ± 0.2	6.1	3.2
	Blackcurrant	33.6 ± 0.1	12.6	6.9
	Gooseberries	38.9 ± 0.2	9.4	5.2
	Quince	42.2 ± 0.2	6.6	3.5
	Grapes	30.3 ± 0.1	15.2	8.6
PACs fraction from sea buckthorn extract			2.3	1.4
Trolox			4.7	4.0

**Table 6:** Antimicrobial activity of lipophilic extracts.

Sample	*E. coli*		*P. aeruginosa*		*S. aureus*		*B. cereus*	
	mg·mL^−1^; CI ≤ 0.3 mg·mL^−1^							
	MIC	MBC	MIC	MBC	MIC	MBC	MIC	MBC
Hexane extract	6.25	6.25	>50	>50	1.56	3.13	6.25	>50
Freon extract	0.78	50	0.78	50	0.39	12.2	3.13	25

## Data Availability

Research data is available upon request from the corresponding author and at https://failiem.lv/u/z69gsx45t (accessed on 2 August 2023).
